# Risk Factors of Postoperative Atrial Fibrillation After Isolated Coronary Artery Bypass Grafting Surgery in the Recent 10 Years: Clinical Analysis of 6229 Patients

**DOI:** 10.1002/clc.24335

**Published:** 2024-10-21

**Authors:** Jia‐Yi Zhou, Jian‐Liang Zhang, Lei Xi, Zhi‐Peng Guo, Xiao‐Cheng Liu, Zhi‐Gang Liu, Qin Yang, Guo‐Wei He

**Affiliations:** ^1^ Department of Cardiovascular Surgery, The Institute of Cardiovascular Diseases & TEDA International Cardiovascular Hospital Tianjin University & Chinese Academy of Medical Sciences Tianjin China; ^2^ Tianjin Key Laboratory of Molecular Regulation of Cardiovascular Diseases and Translational Medicine Tianjin China; ^3^ Division of Cardiothoracic Surgery, Department of Surgery Oregon Health and Science University Portland Oregon USA

**Keywords:** coronary artery bypass grafting, postoperative atrial fibrillation, postoperative complication, risk factor

## Abstract

**Background:**

Postoperative atrial fibrillation (POAF) is a common complication after coronary artery bypass grafting (CABG) that prolongs hospitalization and increases expenses.

**Hypothesis:**

Perioperative risk factors may predict POAF.

**Methods:**

From March 2015 to January 2023, 6229 patients who underwent isolated CABG and were in sinus rhythm before CABG were included in this retrospective study. The preoperative and postoperative variants of patients were collected and analyzed by univariate analyses between the patients with and without POAF. Multivariate logistic regression analysis was then used to study the independent risk factors for POAF.

**Results:**

The incidence of POAF in this group of patients was 30.94%. Univariate analyses demonstrated that age (*p* < 0.001), hypertension (*p* < 0.001), smoking (*p* < 0.05), cardiopulmonary bypass (CPB) time (*p* < 0.01), and ejection fraction (EF, *p* < 0.01) were the risk factors for POAF. Multivariate logistic regression analysis determined the independent risk factors associated with POAF were old age (odds ratio [OR] = 1.062, *p* = 0.000) and low EF (OR = 0.980; *p* = 0.008).

**Conclusions:**

In the current era, after isolated CABG surgery, there is still a quite high incidence of POAF (30.94% in this group of CABG patients). The main risk factors correlating to POAF include age, hypertension, smoking, CPB time, and EF. Among these factors, multivariate analysis identified old age and low EF as the independent risk factors associated with POAF. Particular care should be taken in the perioperative period for these patients in the prevention of POAF.

## Graphical abstract

A graphical abstract demonstrating the findings of this study. In 6229 patients in sinus rhythm undergoing isolated CABG, elderly age and low ejection fraction were identified as independent risk factors for new‐onset postoperative atrial fibrillation.

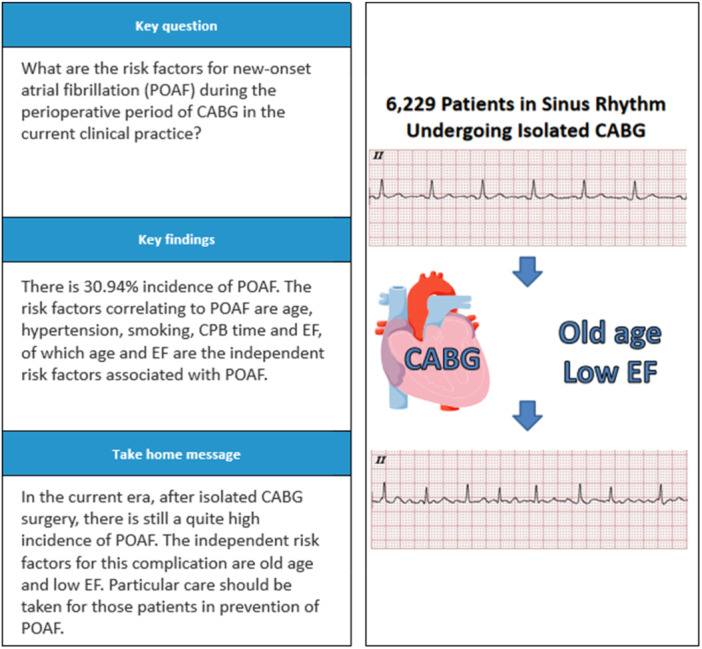

**Abbreviations**
AF: atrial fibrillation
BMI: body mass index
CABG: coronary artery bypass grafting
CPB: cardiopulmonary bypass
EF: ejection fraction
LADs: left atrium systolic diameter
LVDd: left ventricular end diastolic diameter
OPCAB: off‐pump coronary artery bypass grafting
POAF: postoperative atrial fibrillation

## 1 Introduction

New‐onset postoperative atrial fibrillation (POAF) is a common complication after coronary artery bypass grafting (CABG) [1], which also leads to a higher late mortality rate [2, 3]. The incidence of POAF was reported from 20% to 50% [4]. Nearly 30% of the patients have a median onset time of 2 days after CABG. It is anticipated that the incidence of POAF will be even higher in the next decade [5, 6]. The risk factors for the development of POAF have been reported in the previous studies but the results are different [7, 8, 9, 10]. In a study, age, low ejection fraction (EF), and right coronary artery disease were reported as risk factors [8]. Another study identified that apart from age ≥ 70 years, other major predictors for POAF after CABG were preoperative stable angina and low cardiac output syndrome following CABG [9]. Further, with the advancement of cardiac surgical techniques, the mortality and morbidity after CABG change from time to time, and the risk factors for POAF after CABG also change. The present study was designed to investigate the risk factors of POAF after isolated CABG in the current era in 6229 patients.

## 2 Materials and Methods

### 2.1 Patient Selection

This retrospective study included patients who underwent isolated CABG surgery with sinus rhythm before the operation from March 2015 to January 2023. The study was approved by the IRB of TEDA International Cardiovascular Hospital, Tianjin University, Tianjin, China. The exclusion criteria were younger than 18 years old, with atrial fibrillation or other arrhythmia before CABG, and CABG combined with other operations. Patients were divided into a POAF group and a non‐POAF group. The demographic characteristics, medical history, and preoperative and intraoperative variables of the patients were obtained from the Hospital Information System (HIS).

### 2.2 Surgical Technique

All CABG procedures were conducted with general endotracheal anesthesia. Surgeons could opt for on‐pump or off‐pump procedures depending on individual cases. For off‐pump coronary artery bypass grafting (OPCAB) patients, pericardial stitches and heart stabilizers were used to expose the coronary arteries, with intracoronary shunts for myocardial protection. Proximal anastomoses were carried out using a partial clamp technique.

In cases where on‐pump procedures were chosen, the standard cardiopulmonary bypass (CPB) technique was followed. CPB was initiated with a two‐stage venous cannula in the right atrium and ascending aortic cannulation. Bypass flow rate was set at 2 L/m^2^/min with the mean systemic pressure maintained between 50 and 80 mmHg. Body temperature was regulated between 32°C and 34°C depending on the duration of on‐pump time. Warm blood cardioplegia (Buckberg) in anterograde and retrograde routes was used for heart arrest and protection. Post distal anastomosis, retrograde cold blood cardioplegia was initiated. Proximal anastomoses were done on the aortic cross‐clamping using a single clamp technique. Magnesium sulfate (2.5 g) was administered into the CPB circuit at 30 min, and calcium chloride was added if calcium levels were low post‐heart resuscitation. Left internal mammary artery grafts were preferred for the left anterior descending artery whenever feasible. In all the cases, complete revascularization was always attempted. The indication for each procedure was made in accordance with the 2012 ACCF/AHA/ACP/AATS/PCNA/SCAI/STS guideline for the diagnosis and management of patients with stable ischemic heart disease [11].

### 2.3 Criteria for POAF

POAF was defined as multiple episodes of AF lasting more than 30 s, recorded by electrocardiogram monitor or continuous wireless rhythm monitoring. It began immediately after surgery or at any time before discharge and required treatment with anti‐AF drugs, usually amiodarone [11]. All patients who did not meet these requirements and had no amiodarone treatment were excluded from the POAF group. The demographic and clinical data were obtained from the medical records and are shown in Table 1.

**Table 1:** Perioperative demographic characteristics and risk factors for POAF in patients undergoing CABG—univariate analysis.

Variable		Non‐POAF (*n* = 4302)	POAF (*n* = 1927)	*p* value
Sex				0.067
Male	4374	2990	1384 (31.64%)	
Female	1855	1312	543 (29.27%)	
Age (years)		61.75 ± 8.64	65.20 ± 7.77	0.000
BMI (No. of patients)		25.44 ± 3.19 (4050)	25.35 ± 3.09 (1800)	0.305
Hypertension				0.000
Y	3921	2640	1281 (32.67%)	
N	1778	1295	483 (27.17%)	
Diabetes				0.809
Y		1437	641	
N		2347	1031	
Peripheral vascular disease				0.445
Y		402	178	
N		619	250	
Smoking				0.027
Y	2127	1428	699 (32.86%)	
N	2723	1910	813 (29.86%)	
CPB				0.081
Y	4415	3018	1397 (31.64%)	
N	1493	1057	436 (29.20%)	
CPB time		93.05 ± 33.63 (2895)	96.22 ± 35.29 (1336)	0.006
Cross‐clamp time		77.10 ± 32.19 (2918)	78.40 ± 28.30 (1337)	0.206
LADs		37.69 ± 4.12 (596)	37.63 ± 4.08 (262)	0.844
LVDd		52.33 ± 14.87 (1218)	51.67 ± 13.46 (666)	0.324
EF		60.82 ± 8.64 (1217)	59.56 ± 9.49 (664)	0.004

Abbreviations: BMI, body mass index; CPB, cardiopulmonary bypass; EF, ejection fraction; LADs, left atrium systolic diameter; LVDd, Left ventricular end diastolic diameter.

### 2.4 Statistical Analysis

All statistical analyses were performed with SPSS 26.0 software (SPSS Inc., Chicago, IL, USA). Measurement data were expressed as the mean ± SD, and enumeration data were expressed as the number of cases (percentage). These data were analyzed using the χ^2^ test or Student's *t* test. Univariate analysis was performed to find possible factors associated with POAF. Any variables that had trends to be associated with POAF (*p* < 0.1) were included in the stepwise multiple logistic regression analysis [12, 13, 14]. Odds ratios (ORs), corresponding 95% confidence intervals (CIs), and associated *p* values were reported, *p* < 0.05 was considered to indicate an independent risk factor.

## 3 Results

### 3.1 Baseline Characteristics

The overall percentage of POAF in this group of CABG patients was 30.94% (1927 patients). The mean age of the patients was 62.82 ± 8.53 years. Data collected included POAF, sex, age, history of disease (e.g., diabetes, hypertension, etc.), smoking, body mass index (BMI), ultrasound indicators (left atrium systolic diameter, left ventricular end diastolic diameter, and EF), preoperative indicators (CPB, CPB time, etc.), the use of amiodarone, and so on. The average number of grafts was 3.59 ± 0.90.

Table 1 demonstrates patient demographic characteristics.

### 3.2 Univariate Analysis of Risk Factors

Univariate analysis identified that age (*p* < 0.001), hypertension (*p* < 0.001), smoking (*p* < 0.05), CPB time (*p* < 0.01), and EF (*p* < 0.01) were the risk factors for POAF.

Interestingly, the incidence of POAF had no significant differences (*p* > 0.05) after on‐pump CABG (1397/4415, 31.64%) or OPCAB (436/1493, 29.20%) (Table 1).

### 3.3 Multivariate Analysis of Risk Factors

Multivariate logistic regression analysis disclosed that the independent risk factors associated with POAF were age (odds ratio [OR] = 1.062, *p* = 0.000) and EF (OR = 0.980; *p* = 0.008) (Table 2).

**Table 2:** Stepwise multiple logistic regression analysis: independent risk factors for POAF after CABG.

Variable	*B*	SE	Wals	Sig.	Exp (*B*)	95% CI	
Sex	0.061	0.163	0.141	0.708	1.063	0.773	1.462
Age (years)	0.061	0.009	46.778	0.000	1.062	1.044	1.081
Hypertension	0.281	0.151	3.475	0.062	1.325	0.986	1.781
Smoking	0.196	0.151	1.675	0.196	1.216	0.904	1.636
CPB time	0.003	0.002	1.474	0.225	1.003	0.998	1.007
EF	−0.020	0.007	7.084	0.008	0.980	0.966	0.995

Abbreviation: EF, ejection fraction.

### 3.4 Analyses on the Subgroups of Age and EF

The results on the independent risk factors age and EF were further analyzed with subgroups.

**Figure 1:**
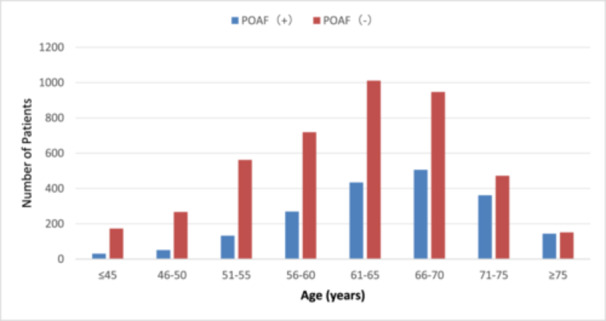
The incidence of POAF in the subgroups of age. The age was divided at every 5‐year interval. The incidence of POAF increases with the advancing age.

When age was further divided into ≤ 45, 46–50, 51–55, 56–60, 61–65, 66–70, 71–75, and ≥ 75 years old, the incidence of POAF was increased with the increased age. It was 14.8%, 16.0%, 19.0%, 27.2%, 30.0%, 34.8%, 43.3%, and 48.8%, respectively (Table 3A and Figure 1).

**Table 3:** The incidence of POAF in subgroups of age (years) and ejection fraction (EF, %).

(A) Age (years)	≤ 45	46–50	51–55	56–60	61–65	66–70	71–75	≥ 75	Total
POAF (+) number of patients	30	51	132	269	434	506	361	144	1927
POAF (−) number of patients	173	267	562	719	1011	947	472	151	4302
Total number of patients	203	318	694	988	1445	1453	833	295	6229
% of POAF (%)	14.8	16.0	19.0	27.2	30.0	34.8	43.3	48.8	30.9

(B) EF (%)	≤ 50	51–55	56–60	61–65	≥ 65	Total
POAF (+) number of patients	100	46	84	323	111	664
POAF (−) number of patients	142	65	137	634	239	1217
Total number of patients	242	111	221	957	350	1881
% of POAF (%)	41.3	41.4	38.0	33.8	31.7	35.3

**Figure 2:**
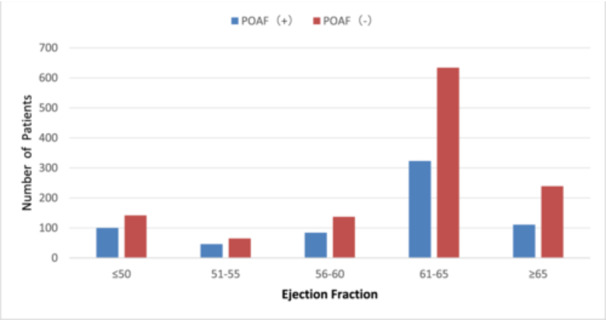
The incidence of POAF in the subgroups of ejection fraction (EF, %). The EF was divided at every 5% interval. The incidence of POAF increases with the decrease of the EF.

Similarly, when EF was further divided into ≤ 50%, 51%–55%, 56%–60%, 61%–65%, and ≥ 65%, the incidence of POAF decreased with the increase of EF. It was 41.3%, 41.4% 38%, 33.8%, and 31.7%, respectively (Table 3B and Figure 2).

## 4 Discussion

The present study reveals that in the current era (1) the incidence of POAF after isolated CABG is still as high as 30.9% (1927/6229) in this single unit; (2) with regard to POAF after isolated CABG procedure, the risk factors by univariate analysis are older age, hypertension, smoking, CPB time, and EF; (3) by multivariate regression analysis, the independent risk factors for POAF are older age and EF; and (4) OPCAB or on‐pump CABG is not an independent risk factor for POAF.

The present study in a large cohort of isolated CABG patients identified old age and low EF as independent risk factors. However, the procedure OPCAB or on‐pump CABG does not have a significant impact on the incidence of POAF.

It has been well known that the prevalence and incidence of AF is associated with age [15]. A report revealed that at the present time, the prevalence of AF (2%) is double than reported in the last decade. Further, the prevalence of AF varies with age and sex. AF is present in 0.12%–0.16% of those younger than 49 years, in 3.7%–4.2% of those aged 60–70 years, and in 10%–17% of those aged 80 years or older [15]. The present study identified old age as an independent risk factor. This is in accordance with the above findings and other previous findings as well [16, 17]. In fact, in our study, the age was 65.20 ± 7.77 versus 61.75 ± 8.64 (*p* = 0.000). To better understand the importance of advanced age in the development of POAF, the patients were further divided into subgroups with every 5‐year intervals (Table 3A and Figure 1). The incidence among the subgroups was from as low as 14.8% in the patients younger than 45 years, gradually increased with advanced age, and reached as high as 48.8% in the patients older than 75 years. These have clearly demonstrated the importance of advanced age in the development of POAF. Therefore, in elderly patients undergoing CABG, careful monitoring of the heart rhythm after the CABG procedure to detect POAF as early as possible is vitally important.

As to sex in correlation with POAF, in the general population, AF occurs more frequently in males, with a male‐to‐female ratio of 1.2:1 [15]. The present study had a trend that the female sex had a protective effect (29.27% in females vs. 31.64% in males, *p* = 0.067) with a male‐to‐female ratio of 1.08:1, but the difference did not reach statistical significance in either univariate or multivariate logistic regression analysis (Tables 1 and 2). Whether this is a reflection of the difference between the “lone” AF in the general population and POAF or due to the different geographical locations needs to be further studied.

Another important factor related to POAF is hypertension. Hypertension was identified as one of the potential risk factors and was entered into a new scoring system for predicting POAF in CABG [17]. In this study, the incidence of POAF was significantly higher in hypertensive patients (1281/3921, 32.67%) than in non‐hypertensive patients (483/1778, 27.17%, *p* = 0.000 in the univariate analysis). Although in the multivariate analysis, the difference between hypertension and non‐hypertension patients did not reach statistical significance (*p* = 0.062), the trend of hypertension as a risk factor is obvious.

In the present study, other common risk factors previously identified such as smoking [18, 19] were also risk factors (32.86% in smoking patients vs. 29.86% in nonsmoking patients, *p* = 0.027). However, similar to hypertension, smoking was not verified as an independent factor in the multivariate logistic regression with a *p* value of 0.196.

An interesting question is whether using CPB is a risk factor for POAF in CABG. In a previous study [20] of 1508 patients who underwent CABG surgery, 686 underwent on‐pump CABG and 822 underwent OPCAB. The results showed that the incidence of POAF was significantly lower in the off‐pump group than that in the on‐pump group. Further analysis revealed that the use of CPB was an independent predictor of POAF after CABG surgery. The authors concluded that OPCAB is associated with a lower incidence of POAF in CABG, meaning that the use of CPB increases the risk of POAF in CABG patients. Furthermore, the duration of CPB is also considered a risk factor for POAF [21].

In comparison to these studies, the present study in a larger CABG cohort compared the incidence of POAF in OPCAB and on‐pump CABG. The results showed that although the incidence of POAF in the OPCAB patients was lower than that in on‐pump CABG patients (29.20% vs. 31.64%), the difference did not reach statistical significance (*p* = 0.081). This demonstrates that OPCAB may have some impact on the reduction of the incidence of POAF as shown in other studies [20]. However, the effect is small and on‐pump is not an independent risk factor for POAF in CABG. Further, in the on‐pump CABG patients, although POAF patients had slightly longer CBP time (96.22 ± 35.29 min) than that in the non‐POAF patients (93.05 ± 33.63 min, Table 1) with statistical significance in the univariate analysis (*p* = 0.006), it was excluded from the multivariate analysis as a significant independent risk factor (Table 2).

One of the major findings from this study was that low EF is an independent risk factor for POAF. Indeed, although the difference in EF between POAF and non‐POAF was not large (Table 1), it was statistically significant (*p* = 0.004). To better understand the importance of EF in the development of POAF, the patients were further divided into subgroups at 5% intervals (Table 3 and Figure 2). The incidence among the subgroups was from as low as 31.7% in the patients with EF ≥ 65%, and gradually increased to as high as 41.3% in the patients with low EF (≤ 50%, Table 3B and Figure 2). These analyses clearly demonstrated the importance of low EF in the development of POAF. Our results are highly in accordance with others. As reported, there is some evidence that echocardiographic parameters can identify patients at greater risk of developing POAF [22, 23, 24]. Patients with reduced EF are at greater risk of developing POAF, possibly because their myocardial damage is more severe. Therefore, EF assessment and individualized treatment plans should be considered in patients with cardiac dysfunction as shown by the low EF in the prevention of POAF.

### 4.1 Limitations of the Study

The present study was a single‐center, retrospective study. To further confirm the risk factors, a multicenter, prospective clinical trial should be performed.

## 5 Conclusions

In the current era, after isolated CABG surgery, there is still a quite high incidence of POAF (30.94% in this group of CABG patients). The main risk factors correlating to POAF include age, hypertension, smoking, CPB time, and EF. Among these factors, multivariate analysis identified old age and low EF as the independent risk factors associated with POAF. Particular care should be taken in the perioperative period for these patients in the prevention of POAF.

## Ethical Statement

The research protocols were approved by the Ethical Committee of TEDA International Cardiovascular Hospital, Tianjin, China (Approval number 2021‐0715‐3) in accordance with the Declaration of Helsinki.

## Conflicts of Interest

The authors declare no conflicts of interest.

## Data Availability

Data used in the current study are available upon reasonable request.
